# Ultrasonic-Assisted Natural Deep Eutectic Solvent Extraction of Proanthocyanidins from *Lycium ruthenicum*: Optimization, Kinetics, and Antioxidant Activity

**DOI:** 10.3390/molecules31142473

**Published:** 2026-07-15

**Authors:** Ying Guo, Ting He, Siyi Wan, Shanmei Tu, Jiaxin Quan, Junkai Ma, Izni Atikah Abd Hamid

**Affiliations:** 1 College of Chemistry and Environmental Engineering, Shiyan Key Laboratory of Biological Resources and Eco-Environmental Protection, Shiyan Key Laboratory of Danjiangkou Reservoir Area’s Aquatic Eco-Environment and Health, Hanjiang Normal University, Shiyan 442000, China; guoying@hjnu.edu.cn (Y.G.); wansiyi@hjnu.edu.cn (S.W.); tushanmei@hjnu.edu.cn (S.T.); 2 Hubei Key Laboratory of Wudang Local Chinese Medicine Research, Department of Chemistry, School of Pharmacy, Hubei University of Medicine, Shiyan 442000, China; 3 Centre for Green Bioprocess Engineering, Faculty of Engineering, Built Environment and Information Technology, SEGi University, Jalan Teknologi, Kota Damansara, Petaling Jaya 47810, Selangor, Malaysia

**Keywords:** *Lycium ruthenicum*, proanthocyanidins, natural deep eutectic solvents, response surface methodology, extraction kinetics, antioxidant activity

## Abstract

Proanthocyanidins, a new class of potent antioxidants, are recognized for their strong in vivo activity and occur in high abundance in *Lycium ruthenicum*. Although the extraction of these compounds from this plant has been extensively investigated, their inherent instability imposes stringent demands on the extraction process. Natural deep eutectic solvents (NADESs) have emerged as attractive alternatives for isolating bioactive constituents from plant materials thanks to their favorable biocompatibility, high extraction efficiency, and environmentally benign nature. Therefore, we have developed an efficient and green NADES-assisted ultrasonic extraction method for recovering proanthocyanidins from *Lycium ruthenicum*. Among the fourteen NADES formulations screened, the choline chloride–glycerol system (1:5 molar ratio, 30% *v*/*v* water) afforded the highest extraction yield. Single-factor experiments, combined with response surface methodology, led to the optimal conditions: solid-to-liquid ratio 1:15 g/mL, ultrasonic power 240 W, temperature 40 °C, and time 30 min. Under these conditions, the proanthocyanidin yield reached 12.19%, markedly outperforming that obtained with conventional methanol extraction. Kinetic analysis indicated that the extraction process conformed to the Logistic model, with coefficients of determination (R^2^) greater than 0.967 across the entire temperature range studied (20–50 °C). In vitro antioxidant assays further revealed that the extract exhibited significant concentration-dependent scavenging activities against •OH, DPPH•, and ABTS^+^• radicals. The IC_50_ values of proanthocyanidin extracts for •OH, DPPH•, and ABTS^+^• were 0.029 mg/mL, 0.56 μg/mL, and 0.011 mg/mL, respectively. Overall, these results establish a sustainable NADES-based protocol for the extraction of proanthocyanidins from *Lycium ruthenicum*, and underscore their promise as natural antioxidants for functional food and pharmaceutical uses.

## 1. Introduction

*Lycium ruthenicum*, a perennial shrub belonging to the Solanaceae family, represents a significant halophytic plant resource distributed across arid regions of Northwest China [1,2]. Historically utilized in traditional Tibetan medicine for treating gynecological disorders and fever, this species has attracted considerable research attention due to its rich phytochemical profile [3]. The dark purple berries contain diverse bioactive constituents, with proanthocyanidins emerging as particularly valuable secondary metabolites distributed throughout roots, stems, leaves, and fruits [4,5].

Proanthocyanidins, polymeric flavonoids constructed from flavan-3-ol monomeric units, exhibit remarkable radical-scavenging capacity attributable to their abundant phenolic hydroxyl moieties. These structural features enable effective termination of free radical chain reactions through hydrogen donation, thereby protecting cellular components from oxidative damage [6]. Contemporary research has established that these compounds demonstrate superior antioxidant efficacy compared to many conventional antioxidants, leading to their classification as potent natural radical scavengers [7]. Beyond their primary antioxidant function, accumulating evidence indicates diverse pharmacological properties including antiproliferative effects against cancer cells, modulation of inflammatory pathways, and maintenance of cardiovascular homeostasis [8,9,10].

Conventional approaches for recovering proanthocyanidins from plant matrices encompass several established techniques, each presenting inherent limitations. Organic solvent extraction requires prolonged processing times and generates substantial chemical waste [11]. Microwave-assisted [12] and supercritical fluid extraction [13] methods demand sophisticated instrumentation and entail considerable operational costs. Enzymatic hydrolysis protocols often suffer from extended reaction periods and strict pH requirements [14,15]. These methodological constraints underscore the necessity for alternative extraction strategies aligned with contemporary green chemistry principles. Ultrasonic-assisted extraction (UAE) exploits cavitation and mechanical forces to rupture cell walls and facilitate the release of polysaccharides [16,17]. This method has extensive application, thanks to its modest energy consumption, eco-friendliness, operational simplicity, and good productivity [18,19]. Environmentally, UAE is frequently ranked among the most promising non-thermal techniques [20], yet the generality of this view remains open to debate.

However, traditional techniques often suffer from complex processing, low extraction efficiency, and high costs. Natural deep eutectic solvents (NADESs) are a novel class of green solvents composed of hydrogen bond acceptors and donors. It is a homogeneous and stable liquids at room temperature [21], exhibit unique physicochemical properties such as low freezing points [22], high viscosity [23], high density [24], excellent solubility [25], and moderate conductivity [26]. They offer several practical advantages, including straightforward preparation, low or negligible toxicity, low cost, ready availability, biodegradability, and feasibility for recovery and reuse. As a promising alternative to conventional organic extraction solvents, NADESs have found broad applications in the chemical, food, and pharmaceutical (notably herbal extraction) industries [27]. Compared to traditional deep eutectic solvents (DESs), NADES components are derived from natural sources, exhibiting lower toxicity and environmental risks [28].

**Scheme 1:**
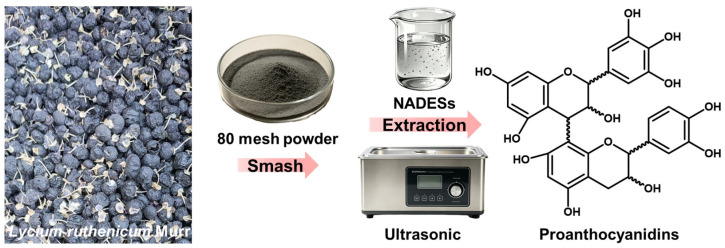
Ultrasonic-assisted extraction of proanthocyanidins from *Lycium ruthenicum* with natural deep eutectic solvents (NADESs).

The integration of NADES with ultrasound-assisted extraction technology offers synergistic benefits for the recovery of bioactive compounds. Ultrasound irradiation generates acoustic cavitation within liquid media, producing localized high-pressure zones and micro-scale turbulence that significantly enhance mass transfer kinetics [29]. These mechanical effects facilitate solvent penetration into the cellular matrix while accelerating the dissolution of target analytes without inducing thermal degradation [30]. Consequently, the combination of green solvent systems with cavitation-assisted extraction represents a rational strategy for developing sustainable separation processes (Scheme 1) [31]. Despite the successful application of NADES-based ultrasound-assisted extraction for recovering various phenolic compounds, systematic investigations of this methodology for proanthocyanidins extraction from *Lycium ruthenicum* remain limited. Therefore, the present study was designed to (i) screen different NADES formulations to identify the optimal solvent system for proanthocyanidins extraction; (ii) employ Response Surface Methodology to systematically optimize the key process parameters affecting extraction efficiency; (iii) establish kinetic models describing the time-dependent proanthocyanidins yield during ultrasonic extraction; and (iv) evaluate the radical scavenging capacity of the obtained extracts through multiple in vitro antioxidant assays. These findings are expected to provide a scientific foundation for the sustainable development and utilization of proanthocyanidins from *Lycium ruthenicum* in functional food and nutraceutical applications.

## 2. Results and Discussion

### 2.1. Screening of the Optimal NADES

Figure S1 illustrates the proanthocyanidin yields obtained with different NADES formulations. Among the tested systems, NADES-1 (choline chloride/glycerol = 1:5, 30%, *v*/*v*) exhibited the highest extraction efficiency. This superior performance can be attributed to two factors: (1) the polarity of the choline chloride/glycerol system closely matches that of proanthocyanidins to enhance solubility [32]; and (2) its relatively low viscosity facilitates mass transfer and compound diffusion during ultrasonic extraction [33]. These results demonstrate that choline chloride/glycerol is the most effective NADES for proanthocyanidins extracted from *Lycium ruthenicum*.

### 2.2. Effect of HBA/HBD Molar Ratio

The experimental procedure was based on the methodologies described by Jesús et al. [34] and Xia et al. [35]. As presented in Figure S2, the proanthocyanidin extraction yield initially increased with rising glycerol (1,2,3-propanetriol) content, reaching a peak of 10.98% at a HBA/HBD molar ratio of 1:5, followed by a sharp decline at higher ratios. This behavior can be explained by the fact that an appropriate amount of glycerol facilitates the dissolution of proanthocyanidins by forming effective hydrogen bonds with the phenolic hydroxyl groups [36]. However, excessive glycerol changes the viscosity of the NADES system, which hinders mass transfer and reduces extraction efficiency [37].

### 2.3. Effect of Water Content

The addition of water to a NADES can significantly reduce its viscosity, increase its polarity, alter its pH, and enhance its conductivity [38]. However, excessive water content may weaken or disrupt the hydrogen bond interactions between NADES components, thereby affecting extraction efficiency [39]. As shown in Figure S3, the extraction efficiency of proanthocyanidins was strongly correlated with the water content of the NADES. When the water content was adjusted to 30% (*v*/*v*), the proanthocyanidins yield was substantially enhanced [40]. This optimal water content provides a balance between reduced viscosity (improving mass transfer) and maintaining the supramolecular structure of the NADES [41].

### 2.4. Single-Factor Experiments for NADES Extraction

#### 2.4.1. Ultrasonic Solid/Liquid Ratio

**Figure 1:**
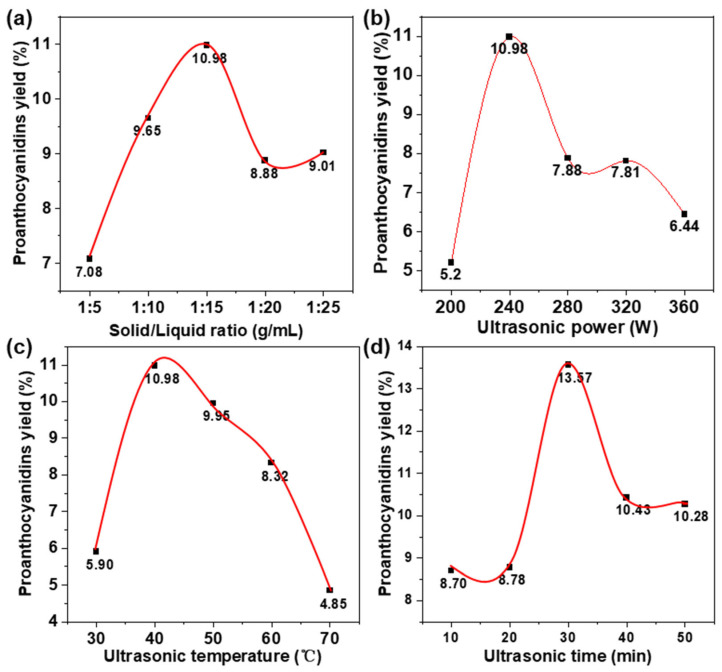
Single-factor experiment for the extraction of proanthocyanidins. (**a**) solid/liquid ratio; (**b**) ultrasonic power; (**c**) ultrasonic temperature; and (**d**) ultrasonic time.

The solid/liquid ratio is a key factor influencing extraction efficiency. As shown in Figure 1a, increasing the solid/liquid ratio from 1:5 to 1:15 (g/mL) led to a marked increase in proanthocyanidin yield. This trend is likely due to the larger volume of NADES, which enhances the contact area between the solvent and the plant material, thereby improving mass transfer [42]. However, when the ratio was further increased to 1:25 (g/mL), the extraction yield decreased. This decline may be attributed to the saturation of proanthocyanidin solubility in the NADES, as well as the co-extraction of undesirable compounds from the sample matrix [43]. Based on these results, a solid/liquid ratio of 1:15 g/mL was selected as optimal for subsequent experiments.

#### 2.4.2. Ultrasonic Power

As shown in Figure 1b, the proanthocyanidins yield increased with ultrasonic power up to 240 W, and it declined beyond 240 W. This trend can be attributed to excessive ultrasonic energy causing degradation of proanthocyanidins, thereby reducing extraction efficiency [44]. Accordingly, an ultrasonic power of 240 W was selected as optimal for subsequent experiments.

#### 2.4.3. Ultrasonic Temperature

Ultrasonic temperature plays a critical role in the extraction process. As shown in Figure 1c, the proanthocyanidins yield increased with rising temperature within the range of 30–40 °C. This can be attributed to enhanced diffusion of bioactive compounds from the plant matrix into the solvent at higher temperatures, as well as reduced viscosity of the NADES, which lowers mass transfer resistance and improves extraction efficiency [45]. However, when the temperature was increased from 40 to 70 °C, a marked decrease in yield was observed. This decline may be due to the thermal degradation of proanthocyanidins at elevated temperatures, as these compounds are heat-sensitive and prone to structural decomposition under prolonged or excessive heating [46]. Therefore, 40 °C was identified as the optimal extraction temperature, and subsequent optimization of the extraction process has been conducted based on this condition.

#### 2.4.4. Ultrasonic Time

The effect of ultrasonic time on proanthocyanidin yield is shown in Figure 1d. Within the range from 10 to 30 min, the yield increased with prolonged extraction time. This can be attributed to enhanced mass transfer and more efficient penetration of the solvent into the plant matrix over time [47]. However, extending the ultrasonic time beyond 30 min resulted in a decline in yield. This decrease is likely due to excessive heat generated during prolonged ultrasonication, which may lead to the thermal degradation of heat-sensitive proanthocyanidins [48]. Accordingly, an ultrasonic time of 30 min was selected as the optimal condition for subsequent experiments.

### 2.5. Response Surface Optimization Experiment

The response surface methodology (RSM) experimental design and corresponding results are presented in Table S3. Regression analysis of the experimental data was performed using Design-Expert 13 software, yielding a quadratic polynomial equation. This equation describes the relationship between proanthocyanidins yield and the four extraction variables:

*Y* = 12.18 + 0.2609*A* − 0.1854*B* + 0.0877*C* + 0.0408*D* + 0.3045*AB* − 0.0142*AC* − 0.2118*AD* − 0.0478*BC* − 0.2105*BD* + 0.0940*CD* − 1.06*A*^2^ − 0.8272*B*^2^ − 0.6675*C*^2^ − 0.5502*D*^2^ (1)

where *Y* is the proanthocyanidins yield (%), and *A*, *B*, *C*, and *D* represent the solid/liquid ratio, ultrasonic power, ultrasonic temperature, and ultrasonic time, respectively.

Analysis of variance (ANOVA) was performed to evaluate the statistical significance of the established regression model (Table 1). The model *F*-value of 23.48 and corresponding *p*-value (<0.01) indicated that the model was highly significant [49]. The goodness-of-fit of the model was further assessed by the lack-of-fit test, which yielded an *F*-value of 3.80 and a *p*-value of 0.0767 (>0.05), suggesting that the lack of fit was not significant [50]. The coefficient of determination (R^2^) and coefficient of variation (CV) were 0.9564 and 4.14%, reflecting strong fitting capability, good repeatability, and stability of the model [51]. Collectively, these statistical indicators confirm that the regression model is well-constructed and provides accurate and reliable predictions.

Regarding the influence of each variable on proanthocyanidin yield, the linear terms of *A* (solid/liquid ratio) and *B* (ultrasonic power) had significant effects (*p* < 0.05), while *C* (ultrasonic temperature) and *D* (ultrasonic time) did not show significant effects (*p* > 0.05). The order of influence of the independent variables on the yield was *A* > *B* > *C* > *D*. All quadratic term coefficients (*A*^2^, *B*^2^, *C*^2^, *D*^2^) exhibited a highly significant impact on the experimental results (*p* < 0.01). Among the interaction effects, only *AB* (the interaction term between solid/liquid ratio and ultrasonic power) had a significant influence on the yield (*p* < 0.05); all other interaction effects were not significant.

**Figure 2:**
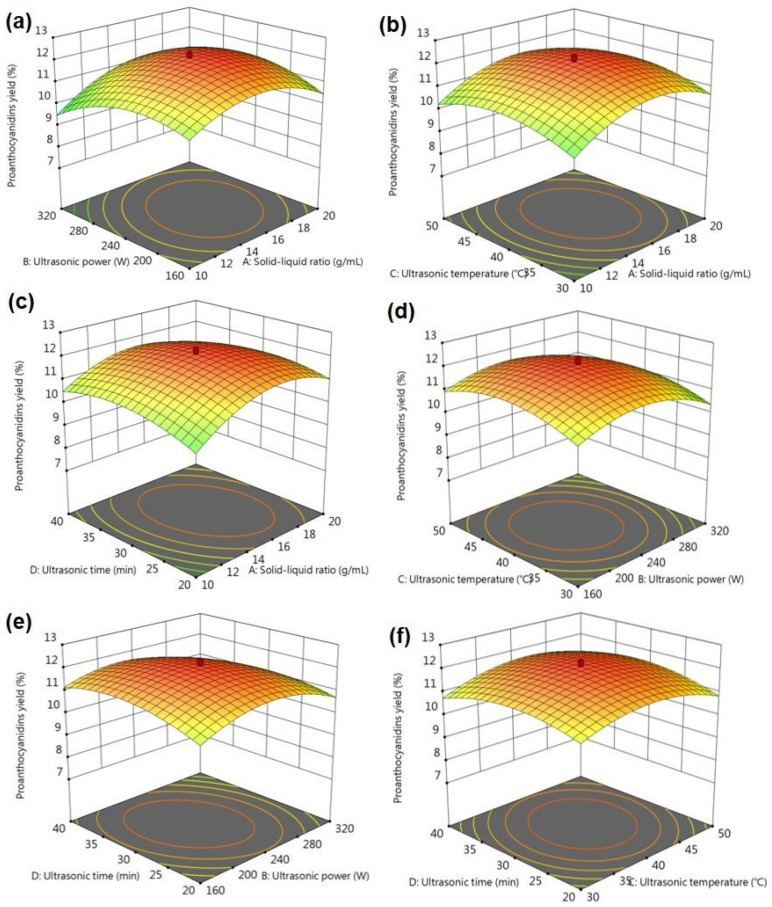
3D response surface plots and contour plots showing the interaction effects of extraction parameters on proanthocyanidins yield: (**a**) solid/liquid ratio and ultrasonic power; (**b**) solid/liquid ratio and ultrasonic temperature; (**c**) solid/liquid ratio and ultrasonic time; (**d**) ultrasonic power and ultrasonic temperature; (**e**) ultrasonic power and ultrasonic time; (**f**) ultrasonic temperature and ultrasonic time.

The interaction effects of factors for proanthocyanidin yield were evaluated by 3D response surface methodology and contour plots (Figure 2). A steeper response surface and elliptical contour lines indicate a significant interaction between factors, while a flatter surface and circular contours suggest weak or negligible interaction [52].

As shown in Figure 2, the AB interaction (solid/liquid ratio and ultrasonic power) exhibited a steep surface and elliptical contour, consistent with the ANOVA result (*p* = 0.0066). At a low solid/liquid ratio, yield first increased and then decreased with increasing ultrasonic power, indicating that an appropriate ratio enhances mass transfer. Maximum yield (12.19%) was achieved at 1:15 g/mL and 240 W, confirming the critical role of both factors and their interaction in process optimization.

### 2.6. Optimization of Extraction Process

Based on the response surface methodology, the optimal conditions for NADES-assisted ultrasonic extraction of proanthocyanidins from *Lycium ruthenicum* were established using Design-Expert 13 software. The predicted optimal parameters were as follows: solid/liquid ratio of 1:15.72 g/mL, ultrasonic power of 232.88 W, ultrasonic temperature of 40.79 °C, and ultrasonic time of 30.42 min, corresponding to a theoretical proanthocyanidins yield of 12.13%. Considering operational feasibility, these conditions were slightly modified to a solid/liquid ratio of 1:15 g/mL, ultrasonic power of 240 W, ultrasonic temperature of 40 °C, and ultrasonic time of 30 min. Verification experiments conducted under these adjusted conditions yielded an average proanthocyanidins yield of 12.19%, which deviated by only 0.54% from the predicted value. The strong correlation between the experimental and predicted values confirms the validity and reliability of the optimized extraction model, demonstrating its practical applicability for proanthocyanidins extraction from *Lycium ruthenicum*. Since UV-Vis absorbance measurements for proanthocyanidin yield can be interfered with other components, we went on to determine the proanthocyanidin content under the optimal conditions by HPLC. These results showed that the extraction yield from the actual sample was 2.23 mg/g, corresponding to a proanthocyanidin yield of 3.35%, which was lower than that obtained by the UV-Vis method.

### 2.7. Kinetic Modeling of the Extraction Process

**Figure 3:**
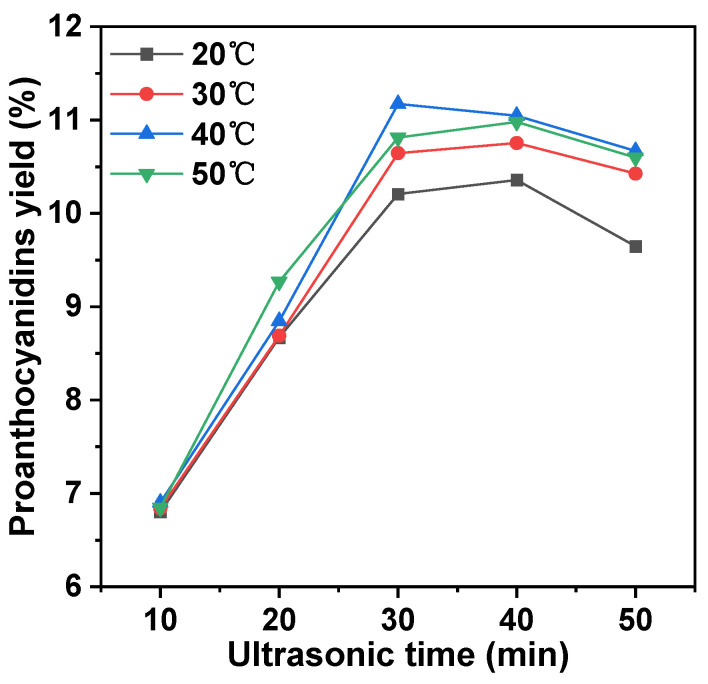
Variation in proanthocyanidins yield with ultrasonic time at different ultrasonic temperatures.

The effect of ultrasonic temperature and time on proanthocyanidins extraction was investigated under the optimized conditions of solid/liquid ratio (1:15 g/mL) and ultrasonic power (240 W) (Figure 3). The experimental data were fitted to various kinetic models, with the resulting kinetic equations and parameters presented in Table 2. The coefficient of determination (R^2^) was employed to evaluate the goodness-of-fit of each model. The Logistic model provided the best description of the relationship between proanthocyanidin yield and extraction time across all tested temperatures (20, 30, 40, and 50 °C), with all R^2^ values exceeding 0.95. These high R^2^ values indicate that the Logistic model accurately captures the extraction kinetics of proanthocyanidins under different temperature conditions [53].

### 2.8. Antioxidant Activity Assays

#### 2.8.1. Hydroxyl Radical Scavenging Activity

**Figure 4:**
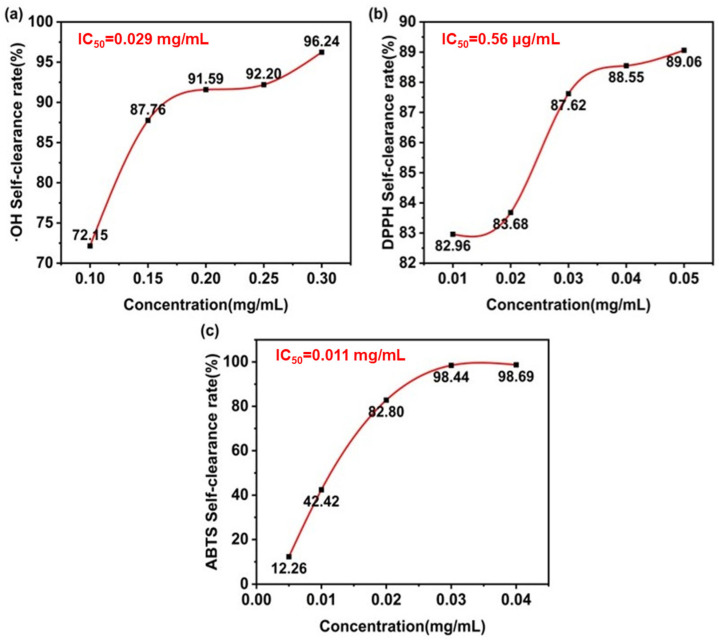
Scavenging effects of the extracted proanthocyanidins on (**a**) •OH; (**b**) DPPH•; and (**c**) ABTS^+^• radicals.

The hydroxyl radical (•OH) scavenging capacity of extracted proanthocyanidins was evaluated at different concentrations (Figure 4a). The proanthocyanidins exhibited a concentration-dependent scavenging effect against •OH radicals. As the concentration increased from 0.10 to 0.30 mg/mL, the scavenging rate progressively rose from 72.15% to 96.24%, demonstrating a strong positive correlation between sample concentration and antioxidant activity [54]. At the highest tested concentration (0.30 mg/mL), the proanthocyanidins achieved excellent •OH scavenging efficiency (96.24%), confirming their potent free radical neutralizing capacity [55].

#### 2.8.2. DPPH• Scavenging Activity

The DPPH radical (DPPH•) scavenging activity of extracted proanthocyanidins is presented in Figure 4b. The scavenging rate increased steadily with increasing sample concentration across the tested range (0.01–0.05 mg/mL) from 82.96% to 89.06%. This concentration-dependent inhibitory effect indicates that extracted proanthocyanidins possess strong DPPH• radical scavenging ability [56]. Notably, even at the lowest concentration (0.01 mg/mL), a high scavenging rate (82.96%) was observed, suggesting the potent radical scavenging nature of these compounds.

#### 2.8.3. ABTS^+^• Scavenging Activity

The ABTS radical (ABTS^+^•) scavenging activity of extracted proanthocyanidins is illustrated in Figure 4c. A positive correlation between scavenging rate and sample concentration was observed. At low concentrations (0.005–0.03 mg/mL), the scavenging rate increased dramatically from 12.26% to 98.44%, indicating efficient radical neutralization. When the concentration reached 0.04 mg/mL, nearly complete scavenging (98.69%) was achieved, demonstrating the exceptional antioxidant capacity of extracted proanthocyanidins at relatively low concentrations. Collectively, these results demonstrate that extracted proanthocyanidins possess strong and concentration-dependent scavenging activities against •OH, DPPH•, and ABTS^+^• radicals, confirming their potential as effective natural antioxidants [57].

Additionally, the IC_50_ values were calculated using Formula (3) with Graphpad Prism software 8.0 based on scavenging rate and mass concentration [58]. The IC_50_ values of the proanthocyanidins extract for •OH, DPPH• and ABTS^+^• were 0.029 mg/mL, 0.56 μg/mL, and 0.011 mg/mL, respectively. These values were lower than those reported for extracts from other plants (Table S5), further confirming the superior antioxidant potency of the proanthocyanidins extracted from *Lycium ruthenicum*.

## 3. Materials and Methods

### 3.1. Materials and Reagents

*Lycium ruthenicum* was provided by Hunan Shennongjinkang Ecological Tea Co., Ltd. (Changsha, China). The samples were dried, ground into powder, and stored in a cool, dry, and dark environment until use.

A proanthocyanidins standard was obtained from Shanghai Yuanye Biotechnology Co., Ltd. (Shanghai, China); vanillin (>98%) and ferrous sulfate (>99%) were supplied by Shanghai Huacheng Industrial Development Co., Ltd. (Shanghai, China); choline chloride (>98%), glucose (>98%), urea (>98%), anhydrous citric acid (>98%), malonic acid (>98%), malic acid (>98%), 1,3-butanediol (>98%), fructose (>98%), potassium persulfate (>99%), salicylic acid (>99%), and 2,2-azino-bis(3-ethylbenzothiazoline-6-sulfonic acid) diammonium salt (ABTS, >98%) were purchased from Anhui Zhesheng Technology Co., Ltd. (Anhui, China); Lactic acid (>98%) and betaine (>98%) were obtained from Shanghai Saen Chemical Technology Co., Ltd. (Shanghai, China); 1,1-Diphenyl-2-picrylhydrazyl (DPPH, >98%) was supplied by Shanghai Macklin Biochemical Technology Co., Ltd. (Shanghai, China); anhydrous ethanol, methanol, and all other reagents used were of analytical grade.

### 3.2. Instruments

The main instruments employed in this study were as follows: a SCQ-7201C digital control ultrasonic cleaning machine (Shanghai Shengyan Ultrasonic Instrument Co., Ltd., Shanghai, China) for ultrasonic-assisted extraction; a HK-2A super thermostatic water bath (Nanjing Nanda Wanhe Technology Co., Ltd., Nanjing, China); a DF-101S heat-collecting thermostatic magnetic stirrer (Jintan Chengxi Lihua Experimental Instrument Factory, Changzhou, China); a DHG-9070A electric constant temperature blast drying oven (Shanghai Jingqi Instrument Co., Ltd., Shanghai, China); a TG16W benchtop high-speed centrifuge (Hunan Pingfan Technology Co., Ltd., Changsha, China); a UV-6100 UV-Vis spectrophotometer (Shanghai Meipuda Instrument Co., Ltd., Shanghai, China); and a GWB-1 water purifier (Beijing Puxian Universal Instrument Co., Ltd., Beijing, China).

### 3.3. Establishment of Proanthocyanidins Standard Curve

**Figure 5:**
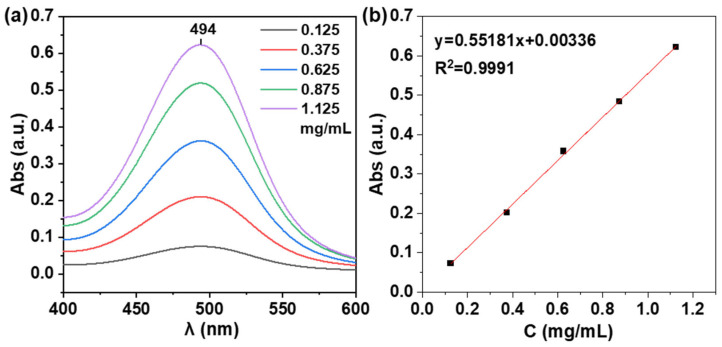
(**a**) Standard curve of proanthocyanidins by UV–Vis experiment; (**b**) linear fit of proanthocyanidins concentration and UV–Vis absorbance.

The proanthocyanidins standard curve was established using the modified vanillin-hydrochloric acid method described by Sun et al. [59]. Briefly, proanthocyanidins standard solutions at various concentrations were prepared, and their absorbance was measured at 494 nm using a UV–Vis spectrophotometer. The standard curve was constructed by plotting absorbance against concentration, yielding the linear regression equation: *y* = 0.5518*x* + 0.0034 (R^2^ = 0.9991), where *y* represents the absorbance and *x* represents the proanthocyanidins concentration (mg/mL). Good linearity was observed within the concentration range of 0.125–1.125 mg/mL (Figure 5).

The proanthocyanidins yield (*W*, %) was calculated according to the following Equation (2):

(2) $$W\;(\%)\;=\;\frac{C\times V\times N}{m\times 1000}\times 100\%$$

where *C* is the proanthocyanidins concentration in the sample solution (mg/mL), determined from the standard curve; *V* is the volume of the sample solution (mL); *N* is the dilution factor; *m* is the mass of *Lycium ruthenicum* powder (g).

### 3.4. Ultrasonic-Assisted Extraction Experiments

According to the method described by Li et al. [60], *Lycium ruthenicum* powder (0.2 g) was accurately weighed and sequentially mixed with distilled water to prepare NADES, and the mixture was thoroughly homogenized. The mixture was subjected to ultrasonic extraction at 40 °C and 240 W for 30 min, followed by centrifugation at 10,000 rpm for 10 min. An aliquot (0.1 mL) of the extract was transferred to a centrifuge tube, to which 5 mL of color developing reagent and 0.9 mL of methanol were successively added. The mixture was incubated in a dark water bath at 30 ± 1 °C for 30 min. Absorbance was measured at 494 nm using methanol as a blank, and the proanthocyanidins yield was calculated according to Equation (2).

### 3.5. Fitting of the Kinetic Model

The kinetic behavior of proanthocyanidins extraction under ultrasonic conditions was investigated following the approach of Susanti et al. [61]. Based on single-factor experiments, proanthocyanidin yields were determined at four temperatures (20, 30, 40, and 50 °C) and five extraction times (10, 20, 30, 40, and 50 min). Three kinetic models-ExpDec1, Allometric1, and Logistic were fitted to the experimental data to describe the extraction process. Model validity was assessed by comparing predicted yields with experimental values at two selected time points (20 and 40 min) for each temperature.

### 3.6. Calculation of IC_50_

The IC_50_ was calculated according to the following formula:

*Y* = (*A*_1_ − *A*_2_)/(1 + (*X*/*X*_0_)^*P*^) + *A*_2_ (3)

where *Y* is the response variable, representing the radical-scavenging activity of the antioxidant. We measure this activity across a range of antioxidant concentrations. The upper and lower asymptotes of the curve are given by *A*_1_ and *A*_2_. *A*_1_ is the maximum response (the highest scavenging rate achieved at a high antioxidant dose), while *A*_2_ is the minimum response (the baseline activity when the antioxidant concentration is extremely low). *X* stands for the antioxidant concentration. *X*_0_ is the concentration at which the response *Y* reaches its half-maximum value, that is, 50% scavenging. In antioxidant assays, this value is commonly referred to as the IC_50_. The parameter *P* determines the shape of the curve; varying P alters the slope and inflection, which allows us to fit the experimental data more accurately. The relationship between antioxidant concentration (*X*) and response (*Y*, i.e., scavenging rate) is described by this nonlinear regression model. We measure the scavenging rate at different concentrations and then fit the data to the equation using a nonlinear regression method—the Levenberg–Marquardt algorithm.

## 4. Conclusions

In this study, a green and efficient ultrasonic-assisted extraction method using NADES was successfully developed for the recovery of proanthocyanidins from *Lycium ruthenicum*. Single-factor experiments combined with response surface methodology optimized the extraction parameters as follows: solid/liquid ratio of 1:15 g/mL, ultrasonic power of 240 W, ultrasonic temperature of 40 °C, and ultrasonic time of 30 min. Under these optimal conditions, the proanthocyanidins yield reached 12.19%. It was superior to that achieved with conventional methanol extraction, demonstrating the potential of NADESs as sustainable alternatives to organic solvents. Kinetic analysis revealed that the extraction process followed the Logistic model, with high coefficients of determination (R^2^ > 0.9672) across all tested temperatures (20–50 °C). Validation experiments confirmed the model’s predictive accuracy, with fitting degrees exceeding 0.934 between experimental and predicted values, establishing a reliable theoretical foundation for process scale-up and control.

Furthermore, the antioxidant potential of the extracted proanthocyanidins was comprehensively evaluated. These results demonstrated significant concentration-dependent scavenging activities against •OH, DPPH•, and ABTS^+^•. Notably, at a concentration of 0.04 mg/mL, the proanthocyanidins achieved 98.69% ABTS radical scavenging. The IC_50_ of the proanthocyanidin extract for •OH, DPPH•, and ABTS^+^• were 0.029 mg/mL, 0.56 μg/mL, and 0.011 mg/mL, respectively. In conclusion, this study establishes an efficient, environmentally friendly NADES-based ultrasonic-assisted extraction protocol for proanthocyanidins from *Lycium ruthenicum*. It also provides systematic insights into the extraction kinetics and antioxidant properties of the recovered compounds. Compared with conventional extraction methods, the NADES system affords higher extraction yields. In addition, the solvent is environmentally friendly and recyclable, thereby providing a more efficient solvent system for the extraction of bioactive constituents from plant materials. This demonstrated bioactivity supports the potential application of extracted proanthocyanidins as natural antioxidants in functional foods and pharmaceutical products.

## Figures and Tables

**Table 1:** ANOVA statistics of the quadratic model for proanthocyanidins extraction efficiency by NADES.

Variance Source	Sum of Squares	Degree of Freedom	Mean Square	*F*-Value	*p*-Value	Significance
Model	52.92	14	3.78	23.48	<0.0001	**
*A*	2.43	1	2.43	15.12	0.0015	**
*B*	0.7585	1	0.7585	4.71	0.0464	*
*C*	0.2179	1	0.2179	1.35	0.2629	
*D*	0.0562	1	0.0562	0.3491	0.5634	
*AB*	1.6	1	1.6	9.92	0.0066	**
*AC*	0.0001	1	0.0001	0.0007	0.9788	
*AD*	0.642	1	0.642	3.99	0.0643	
*BC*	0.0211	1	0.0211	0.1311	0.7224	
*BD*	0.634	1	0.634	3.94	0.0658	
*CD*	0.1777	1	0.1777	1.1	0.3101	
*A^2^*	26.24	1	26.24	162.96	<0.0001	**
*B^2^*	18.25	1	18.25	113.35	<0.0001	**
*C^2^*	11.8	1	11.8	73.31	<0.0001	**
*D^2^*	7.96	1	7.96	49.43	<0.0001	**
Residual	2.42	15	0.161			
Fictitious term	2.13	10	0.2135	3.8	0.0767	
Pure error	0.2806	5	0.0561			
Sum	55.34	29				
R^2^	0.9564					
$R_{adj}^{2}$	0.9156					
$R_{pre}^{2}$	0.7705					
C.V.%	4.14					

Note: ** indicates a statistically significant difference (*p* ˂ 0.01); * indicates a significant difference (*p* ˂ 0.05).

**Table 2:** Fitting parameters of kinetic models for proanthocyanidin extraction at different ultrasonic temperatures.

Model Name and Equation	Model Fitting Parameters	Ultrasonic Temperature (°C)			
		20	30	40	50
ExpDec1 $y=y_{0}+A_{1}e^{-x/t_{1}}$	*y*_0_	10.1724	10.9151	11.2142	10.9751
	*A*_1_	−10.0436	−9.6312	−10.6947	−11.9626
	*t*_1_	9.3012	11.9256	11.3104	9.4913
	R^2^	0.9260	0.9481	0.9252	0.9736
Allometric1 *y* = *ax^b^*	*a*	4.3383	3.9135	3.9132	4.0752
	*b*	0.2261	0.2678	0.2758	0.2629
	R^2^	0.7831	0.8605	0.8235	0.8425
Logistic $y=\frac{A_{1}-A_{2}}{1+(\frac{x}{x_{0}}{)}^{p}}+A_{2}$	*A*_1_	6.8015	6.8382	6.9019	6.8472
	*A*_2_	10.0702	10.6080	10.9626	10.7964
	*x*_0_	19.8661	20.0152	20.0350	19.7731
	*p*	43.1301	47.8579	49.1052	40.3095
	R^2^	0.9672	0.9952	0.9898	0.9939

## Data Availability

The original contributions presented in this study are included in the article/Supplementary Material. Further inquiries can be directed to the corresponding author.

## Supplementary Materials

The following supporting information can be downloaded at: https://www.mdpi.com/article/10.3390/molecules31142473/s1, Table S1: composition and physical properties of the prepared NADES systems; Figure S1: Effect of different NADES formulations on the extraction yield of proanthocyanidins from *Lycium ruthenicum* (Extraction conditions: solid/liquid ratio 1:15 g/mL, ultrasonic extraction at 40 °C and 240 W for 30 min); Figure S2: The extraction yield of proanthocyanidins from *Lycium ruthenicum* by NADES with different ratios of HBA and HBD; Figure S3: Effect of water content on the extraction yield of proanthocyanidins from *Lycium ruthenicum*; Figure S4: Fourier Transform infrared spectroscopy (FTIR) for the NADES (choline chloride /glycerol) with different water contents; Table S2: Factors and levels studied using Central Composite Design (CCD); Table S3: Experimental design and results of Central Composite Design (CCD); Table S4: Verification of extraction kinetic model of proanthocyanidins; Figure S5: (a) Standard curve of proanthocyanidins by HPLC experiments; (b) the actual extract proanthocyanidins from *Lycium ruthenicum* was tested by HPLC; Table S5: Studies on the antioxidant activities of the activities extracts from various plant species. References [62,63,64,65,66,67,68,69] are cited in Supplementary Materials.

## Abbreviations

The following abbreviations are used in this manuscript:

NADES	Natural Deep Eutectic Solvent
HBA	Hydrogen-bond acceptor
HBD	Hydrogen-bond donor
RSM	Response Surface Methodology
DPPH	2,2-diphenyl-1-picrylhydrazyl
ABTS	2,2′-azino-bis(3-ethylbenzothiazoline-6- sulfonic acid

## References

[1] Li W., Rao S., Du C., Liu L., Dai G., Chen J. (2022). Strategies used by two goji species, *Lycium ruthenicum* and *Lycium barbarum*, to defend against salt stress. Sci. Hortic..

[2] Gong H., Rehman F., Ma Y., Biao A., Zeng S.H., Yang T.S., Huang J.G., Li Z., Wu D.P., Wang Y. (2022). Germplasm resources and strategy for genetic breeding of *Lycium* species: A review. Front. Plant Sci..

[3] Luo Z., Yu G., Chen X., Liu Y., Zhou Y., Wang G.P., Shi Y.Y. (2020). Integrated phytochemical analysis based on UHPLC-LTQ-Orbitrap and network pharmacology approaches to explore the potential mechanism of *Lycium ruthenicum Murr*. for ameliorating Alzheimer’s disease. Food Funct..

[4] Li D., Jin J. (2025). Regulatory effects of *Lycium barbarum* polysaccharides on immune function and their application prospects. Front. Immunol..

[5] Zhao Y., Jiang C., Lu J., Sun Y.H., Cui Y.Y. (2023). Research progress of proanthocyanidins and anthocyanidins. Phytother. Res..

[6] Rauf A., Imran M., Abu-Izneid T., Ul-Haq I., Patel S., Pan X.D., Naz S., Silva A.S., Saeed F., Suleria H.A.R. (2019). Proanthocyanidins: A comprehensive review. Biomed. Pharmacother..

[7] Chen R., Zhou X., Deng Q., Yang M.H., Li S.Y., Zhang Q.R., Sun Y., Chen H.G. (2024). Extraction, structural characterization and biological activities of polysaccharides from *mulberry* leaves: A review. Int. J. Biol. Macromol..

[8] Tang W.M., Chan E., Kwok C.Y., Lee Y.K., Wu J.H., Wan C.W., Chan R.Y.K., Yu P.H.F., Chan S.W. (2012). A review of the anticancer and immunomodulatory effects of *Lycium barbarum* fruit. Inflammopharmacology.

[9] Bagchi D., Bagchi M., Stohs S.J., Das D.K., Ray S.D., Kuszynski C.A., Joshi S.S., Pruess H.G. (2000). Free radicals and grape seed proanthocyanidin extract: Importance in human health and disease prevention. Toxicology.

[10] Kruger M.J., Davies N., Myburgh K.H., Lecour S. (2014). Proanthocyanidins, anthocyanins and cardiovascular diseases. Food Res. Int..

[11] Monrad J.K., Howard L.R., King J.W., Srinivas K., Mauromoustakos A. (2010). Subcritical solvent extraction of procyanidins from dried *red grape* pomace. J. Agric. Food Chem..

[12] Liu Z., Li H., Qi Y., Zhu Z., Huang D., Zhang K., Pan J., Wen L., Zou Z.G. (2021). *Cinnamomum camphora* leaves as a source of proanthocyanidins separated using microwave-assisted extraction method and evaluation of their antioxidant activity in vitro. Arab. J. Chem..

[13] Yilmaz E.E., Özvural E.B., Vural H. (2011). Extraction and identification of proanthocyanidins from *grape* seed (Vitis vinifera) using supercritical carbon dioxide. J. Supercrit. Fluids.

[14] Fernández K., Vega M., Aspé E. (2015). An enzymatic extraction of proanthocyanidins from *País grape* seeds and skins. Food Chem..

[15] Liang L., Liu Y., Wu L., Weng L., Qiu H.H., Zhong W.T., Meng F.X. (2024). Advances in extraction protocols, degradation methods, and bioactivities of proanthocyanidins. Molecules.

[16] Zhang D., Huang D., Zhang Y., Lu Y., Huang S., Gong G., Li L. (2023). Ultrasonic assisted far infrared drying characteristics and energy consumption of *ginger* slices. Ultrason. Sonochem..

[17] Li J., Ye G., Zhou Z., Wang J., Wang J., Ju T., Cifuentes A., Ibanez D., Baranenko D., Zhao H. (2025). Ultrasound-assisted extraction of polysaccharide from *Allium chinense G. Don epidermal* waste: Evaluation of extraction mechanism, physicochemical properties, and bioactivities. Ultrason. Sonochem..

[18] Wang C., Li J., Cao Y., Huang J., Lin H., Zhao T., Liu L., Shen P., McClements D., Chen J. (2023). Extraction and characterization of pectic polysaccharides from *Choerospondias axillaris* peels: Comparison of hot water and ultrasound-assisted extraction methods. Food Chem..

[19] Li J., Ye G., Wang J., Gong T., Wang J., Zeng D., Cifuentes A., Ibanez E., Zhao H., Lu W. (2025). Recent advances in pressurized hot water extraction/modification of polysaccharides: Structure, physicochemical properties, bioactivities, and applications. Compr. Rev. Food Sci. Food Saf..

[20] Niknam S.M., Kashaninejad M., Escudero I., Sanz M.T., Beltran S., Benito J.M. (2021). Valorization of *olive* mill solid residue through ultrasound-assisted extraction and phenolics recovery by adsorption process. J. Clean. Prod..

[21] Spaggiari C., Carbonell-Rozas L., Zuilhof H., Costantino G., Righetti L. (2025). Structural elucidation and long-term stability of synthesized NADES: A detailed physicochemical analysis. J. Mol. Liq..

[22] Chemat F., Vian M.A., Cravotto G. (2012). Green extraction of natural products: Concept and principles. Int. J. Mol. Sci..

[23] Freitas D.S., Ribeiro A., Cavaco-Paulo A., Silva C. (2025). The versatility of NADES across applications. Molecules.

[24] Jia L.Y., Huang X., Zhao W., Wang H., Jing X. (2020). An effervescence tablet-assisted microextraction based on the solidification of deep eutectic solvents for the determination of strobilurin fungicides in water, juice, wine, and vinegar samples by HPLC. Food Chem..

[25] Dai Y., van Spronsen J., Witkamp G.J., Verpoorte R., Choi Y.H. (2013). Natural deep eutectic solvents as new potential media for green technology. Anal. Chim. Acta.

[26] Smirnov M.A., Nikolaeva A.L., Vorobiov V.K., Bobrova N.V., Abalov I.V., Smirnov A.V., Sokolova M.P. (2020). Ionic conductivity and structure of chitosan films modified with lactic acid-choline chloride NADES. Polymers.

[27] Choi Y.H., Spronsen J.V., Dai Y.T., Verberne M., Hollmann F., Arends I.W.C.E., Witkamp G., Verpoorte R. (2011). Are natural deep eutectic solvents the missing link in understanding cellular metabolism and physiology?. Plant Physiol..

[28] Hikmawanti N.P.E., Ramadon D., Jantan I., Mun’im A. (2021). Natural deep eutectic solvents (NADES): Phytochemical extraction performance enhancer for pharmaceutical and nutraceutical product development. Plants.

[29] Li S.S., Wang G.Y., Zhao J.J., Ou P.G., Yao Q.P., Wang W. (2024). Ultrasound-assisted extraction of phenolic compounds from *celtuce (Lactuca sativa var. augustana)* leaves using natural deep eutectic solvents (NADES): Process optimization and extraction mechanism research. Molecules.

[30] Ilić N.E., Stupar A., Matic M., Đerić M., Mišan A., Pojić M., Teslić N., Brunet J.Č., Mandić A. (2025). Enhancing bioactive compound extraction from *hemp sprouts*: Synergistic effects of NADES and ultrasound. Ind. Crops Prod..

[31] Pereira T.C., Souza V.P., Padilha A.P.F., Duarte F.A., Flores E.M. (2025). Trends and perspectives on the ultrasound-assisted extraction of bioactive compounds using natural deep eutectic solvents. Curr. Opin. Chem. Eng..

[32] Neto R.T., Santos S.A.O., Oliveira J., Silvestre A.J.D. (2020). Tuning of proanthocyanidin extract’s composition through quaternary eutectic solvents extraction. Antioxidants.

[33] Faraone A., Wagle D.V., Baker G.A., Novak E.C., Ohl M., Reuter D., Lunkenheimer P., Loidl A., Mamontov E. (2018). Glycerol hydrogen-bonding network dominates structure and collective dynamics in a deep eutectic solvent. J. Phys. Chem. B.

[34] Jesús D.M.B., Carmen D.M.C., Basilio J.H., Bastidas-Bastidas P.D.J., Gutiérrez-Grijalva E.P., León-Félix J., Angulo-Escalante M.Á. (2023). Green extracts and UPLC-TQS-MS/MS profiling of flavonoids from *Mexican oregano (Lippia graveolens)* using natural deep eutectic solvents/ultrasound-assisted and supercritical fluids. Plants.

[35] Xia B.H., Yan D., Bai Y.B., Xie J.C., Cao Y., Liao D.F., Lin L.M. (2015). Determination of phenolic acids in *Prunella vulgaris L.*: A safe and green extraction method using alcohol-based deep eutectic solvents. Anal. Methods.

[36] Jovanović M.S., Krgović N., Radan M., Ćujić-Nikolić N., Mudrić J., Lazarević Z., Šavikin K. (2023). Natural deep eutectic solvents combined with cyclodextrins: A novel strategy for *chokeberry* anthocyanins extraction. Food Chem..

[37] Jauregi P., Esnal-Yeregi L., Labidi J. (2024). Natural deep eutectic solvents (NADES) for the extraction of bioactives: Emerging opportunities in biorefinery applications. PeerJ Anal. Chem..

[38] Dai Y., Witkamp G.J., Verpoorte R., Choi Y.H. (2015). Tailoring properties of natural deep eutectic solvents with water to facilitate their applications. Food Chem..

[39] Kaur S., Kumari M., Kashyap H.K. (2020). Microstructure of deep eutectic solvents: Current understanding and challenges. J. Phys. Chem. B.

[40] Singh L., Singh B., Bhatt I.D. (2024). NADES-based extraction optimization and enrichment of cyanidin-3-O-galactoside from *Rhododendron arboreum Sm*.: Kinetics and thermodynamics insights. Food Chem..

[41] Albertini B., Bertoni S., Sangiorgi S., Nucci G., Passerini N., Mezzina E. (2023). NaDES as a green technological approach for the solubility improvement of *BCS class II APIs*: An insight into the molecular interactions. Int. J. Pharm..

[42] Qi H.T., Fu W.J., Liu Y.J., Bai J.Q., Wang R.L., Zou G.M., Shen H.Y., Cai Y.Y., Luo A.W. (2025). Electron beam irradiation coupled ultrasound-assisted natural deep eutectic solvents extraction: A green and efficient extraction strategy for proanthocyanidin from *walnut* green husk. Food Chem..

[43] Viñas-Ospino A., Gómez-Urios C., López-Malo D., Penadés-Soler A., Frígola A., Esteve M.J., Blesa J. (2021). Green Extraction of Flavonoids from Orange Peels Using Deep Eutectic Solvents. Biol. Life Sci. Forum.

[44] Ma Y.Q., Ye X.Q., Fang Z.X., Chen J.C., Xu G.H., Liu D.H. (2008). Phenolic compounds and antioxidant activity of extracts from ultrasonic treatment of *Satsuma Mandarin (Citrus Unshiu Marc.)* Peels. J. Agric. Food Chem..

[45] Ramić M., Zeković Z., Vidović S., Vladić J., Cvejin A., Pavlić B. (2015). Modeling and optimization of ultrasound-assisted extraction of polyphenolic compounds from *Aronia melanocarpa* by-products from filter-tea factory. Ultrason. Sonochem..

[46] Xu Z., Wei L.H., Ge Z.Z., Zhu W., Li C.M. (2015). Comparison of the degradation kinetics of A-type and B-type proanthocyanidins dimers as a function of pH and temperature. Eur. Food Res. Technol..

[47] Cui J., Fang D., Tian X., Peng J., Chen D., Xu S.J., Ma L. (2023). Sustainable conversion of *cottonseed* hulls to valuable proanthocyanidins through ultrasound-assisted deep eutectic solvent extraction. Ultrason. Sonochem..

[48] Liu X., Le Bourvellec C., Guyot S., Renard C.M.G.C. (2021). Reactivity of flavanols: Their fate in physical food processing and recent advances in their analysis by depolymerization. Compr. Rev. Food Sci. Food Saf..

[49] Liang H., Wang W.C., Xu J., Zhang Q., Shen Z.L., Zeng Z., Li Q.Y. (2017). Optimization of ionic liquid-based microwave-assisted extraction technique for curcuminoids from *Curcuma longa* L.. Food Bioprod. Process..

[50] Rheem S. (2023). Optimizing food processing through a new approach to response surface methodology. Food Sci. Anim. Resour..

[51] Rezaei M., Pourang N., Moradi A.M. (2022). Removal of lead from aqueous solutions using three biosorbents of aquatic origin with the emphasis on the affective factors. Sci. Rep..

[52] Abu M.L., Nooh H.M., Oslan S.N., Salleh A.B. (2017). Optimization of physical conditions for the production of thermostable T1 lipase in Pichia guilliermondii strain SO using response surface methodology. BMC Biotechnol..

[53] Natolino A., Da Porto C. (2020). Kinetic models for conventional and ultrasound assistant extraction of polyphenols from defatted fresh and distilled *grape* marc and its main components skins and seeds. Chem. Eng. Res. Des..

[54] Liu X.K., Wang Y., Ge W., Cai G.Z., Guo Y.L., Gong J.Y. (2022). Spectrum–effect relationship Between ultra-high-performance liquid chromatography fingerprints and antioxidant activities of *Lophatherum gracile Brongn*. Food Sci. Nutr..

[55] Bors W., Michel C. (1999). Antioxidant capacity of flavanols and gallate esters: Pulse radiolysis studies. Free Radic. Biol. Med..

[56] Chen S.S., Hu N., Wang H.L., Wu Y.N., Li G.L. (2022). Bioactivity-guided isolation of the major anthocyanin from *Lycium ruthenicum Murr*. fruit and its antioxidant activity and neuroprotective effects in vitro and in vivo. Food Funct..

[57] Shen M.L., Liu K., Liang Y.F., Liu G.X., Sang J., Li C.Q. (2020). Extraction optimization and purification of anthocyanins from *Lycium ruthenicum Murr*. and evaluation of tyrosinase inhibitory activity of the anthocyanins. J. Food Sci..

[58] Bag A., Ghorai P.K. (2016). Development of quantum chemical method to calculate half maximal inhibitory concentration (IC 50). Mol. Inform..

[59] Sun B.S., Ricardo-da-Silva J.M., Spranger I. (1998). Critical factors of vanillin assay for catechins and proanthocyanidins. J. Agric. Food Chem..

[60] Li M.H., Rao C., Wang C.X., Cui X.M., Xiong Y. (2024). Natural deep eutectic solvents-based selective extraction of saponins from *Panax notoginseng*: Process optimization, chemical profiling, and bioactivities evaluation. Sep. Purif. Technol..

[61] Susanti D.Y., Sediawan W.B., Fahrurrozi M., Hidayat M. (2021). The effects of ultrasound wave on the extraction of proanthocyanidins from *red sorghum* grain using green solvent and a kinetics model of the extraction. Key Eng. Mater..

[62] Han X., Zhou Q., Gao Z., Xu G.B., Chen H., Chitrakar B., Sun Y.S., Zhao W., Lin X., Zhou K.X. (2023). Characterization of procyanidin extracts from hawthorn (*Crataegus pinnatifida*) in human colorectal adenocarcinoma cell line Caco-2, simulated digestion, and fermentation identified unique and novel prebiotic properties. Food Res. Int..

[63] Delgado M.C., Merino J., Estevez M., Silvestre A.J.D. (2021). Impact of eutectic solvents utilization in the microwave assisted extraction of proanthocyanidins from grape pomace. Molecules.

[64] Bajpai S., Gupta S.K., Dey A., Jha M.K., Bajpai V., Joshi S., Gupta A. (2012). Application of central composite design approach for removal of chromium (VI) from aqueous solution using weakly anionic resin: Modeling, optimization, and study of interactive variables. J. Hazard. Mater..

[65] Xiao J., Li S.Y., Sui Y., Li X.P., Wu Q., Zhang R.F., Zhang M.W., Xie B.J., Sun Z.D. (2015). In vitro antioxidant activities of proanthocyanidins extracted from the lotus seedpod and ameliorative effects on learning and memory impairment in scopolamine-induced amnesia mice. Food Sci. Biotechnol..

[66] Lu D., Wang L.J., Zhang W., Guo B.H., Lv Y.G. (2022). Study on the antioxidant ability of procyanidins and their complexes. E3S Web Conf..

[67] Aung T., Bibat M.A.D., Zhao C.C., Eun J.B. (2020). Bioactive compounds and antioxidant activities of *Quercus salicina* Blume extract. Food Sci. Biotechnol..

[68] Yang H.B., Xu P.L., Song W., Zhai X.Q. (2017). Anti-tyrosinase and antioxidant activity of proanthocyanidins from Cinnamomum camphora. Int. J. Food Prop..

[69] Yu L., Feng S., Song Y., Bi J., Gao Y., Wang L.H., Jiang C., Wang M.Q. (2025). Exploring the extraction, antioxidant activities and its stabilities of peanut skins crude proanthocyanidins extract. Sci. Rep..

