# Supplementary material for: Ultrasonic-Assisted Natural Deep Eutectic Solvent Extraction of Proanthocyanidins from Lycium ruthenicum: Optimization, Kinetics, and Antioxidant Activity
Source: Molecules. 2026 Jul 15;31(14):2473. doi: 10.3390/molecules31142473 (PMC13414100; doi:10.3390/molecules31142473)
Supplement: Supplementary file 1 [file molecules-31-02473-s001.zip › molecules-4309355-supplementary.pdf]

Supporting Information

Ultrasonic-assisted Natural Deep Eutectic Solvent Extraction of Proanthocyanidins from *Lycium ruthenicum*: Optimization, Kinetics, and Antioxidant Activity

Ying Guo[1], Ting He[1], Siyi Wan[1], Shanmei Tu[1], Jiaxin Quan[1,*], Junkai Ma[2,*] and Izni Atikah Abd Hamid[3,*]

[1] College of Chemistry and Environmental Engineering, Shiyan Key Laboratory of Biological Resources and Eco-Environmental Protection, Shiyan Key Laboratory of Danjiangkou Reservoir Area's Aquatic Eco-Environment and Health, Hanjiang Normal University, Shiyan 442000, China

[2] Hubei Key Laboratory of Wudang Local Chinese Medicine Research, Department of Chemistry, School of Pharmacy Hubei University of Medicine, Shiyan 442000, China

[3] Centre for Green Bioprocess Engineering, Faculty of Engineering, Built Environment and Information Technology, SEGi University, Jalan Teknologi, Kota Damansara, 47810 Petaling Jaya, Selangor, Malaysia

1. NADES experiments

1.1. Preparation of NADES

Following previously reported methods [0**Error! Reference source not found.**, 14 different Natural Deep Eutectic Solvents(NADES) formulations were prepared for screening. Hydrogen bond acceptors (HBAs) and hydrogen bond donors (HBDs) were mixed at specific molar ratios and heated in a 90 °C oil bath with continuous stirring until a uniform, clear, and stable solution was formed. The extraction efficiency of proanthocyanidins from *Lycium ruthenicum* powder was evaluated using the 14 NADES formulations. **Table S1** summarizes the composition and physical state of the prepared NADES systems. Based on their stability and homogeneity, suitable NADES were selected for further extraction experiments.

Table S1. Composition and physical properties of the prepared NADES systems.

Number	NADES description	HBA/HBD	Appearance at room temperature	Water content
NADES-1	Choline chloride/glycerol	1:1	Crystallized	30%
		1:2	Colorless and transparent liquid with low viscosity	30%
NADES-2	Choline chloride/glucose	1:1	Insoluble, unable to form a homogeneous clear solution	30%
		1:2	Yellow transparent liquid with high viscosity	30%
NADES-3	Choline chloride/urea	1:1	Crystallized	30%
		1:2	Colorless and transparent liquid with low viscosity	30%
NADES-4	Choline chloride/ anhydrous citric acid	1:1	Insoluble, unable to form a homogeneous clear solution	30%
		1:2	Insoluble, unable to form a homogeneous clear solution	30%
		2:1	Insoluble, unable to form a homogeneous clear solution	30%
NADES-5	Choline chloride/ propionic acid	1:1	Colorless and transparent liquid with low viscosity	30%
NADES-6	Choline chloride/ L-lactic acid	1:1	Crystallized	30%
		1:2	Colorless and transparent liquid with low viscosity	30%
NADES-7	Choline chloride/ 1,3-butanediol	1:1	Crystallized	30%
		1:2	Colorless and transparent liquid with low viscosity	30%
NADES-8	Choline chloride/	1:1	Crystallized after adding water	30%

	oxalic Acid	2:1	Colorless and transparent liquid with low viscosity	30%
NADES-9	Choline chloride/ DL-malic acid	1:1	Highly viscous liquid	30%
NADES-10	Choline chloride/ D-fructose	1:1	Colorless and transparent liquid with low viscosity	30%
NADES-11	Betaine/DL-malic acid	1:1	Colorless and transparent liquid with low viscosity	30%
NADES-12	Betaine/glycerol	1:1	Insoluble, unable to form a homogeneous clear solution	30%
NADES-13	Betaine/ urea	1:1	Insoluble, unable to form a homogeneous clear solution	30%
NADES-14	Betaine/L-lactic acid	1:1	Yellowish transparent liquid with moderate viscosity	30%

1.2. Screening of NADES

Based on the Ultrasonic-assisted extraction method, different NADES systems were evaluated for their extraction efficiency of proanthocyanidins from *Lycium ruthenicum*. The NADES system that gave the highest proanthocyanidins yield was selected as the optimal one (**Figure S1**).

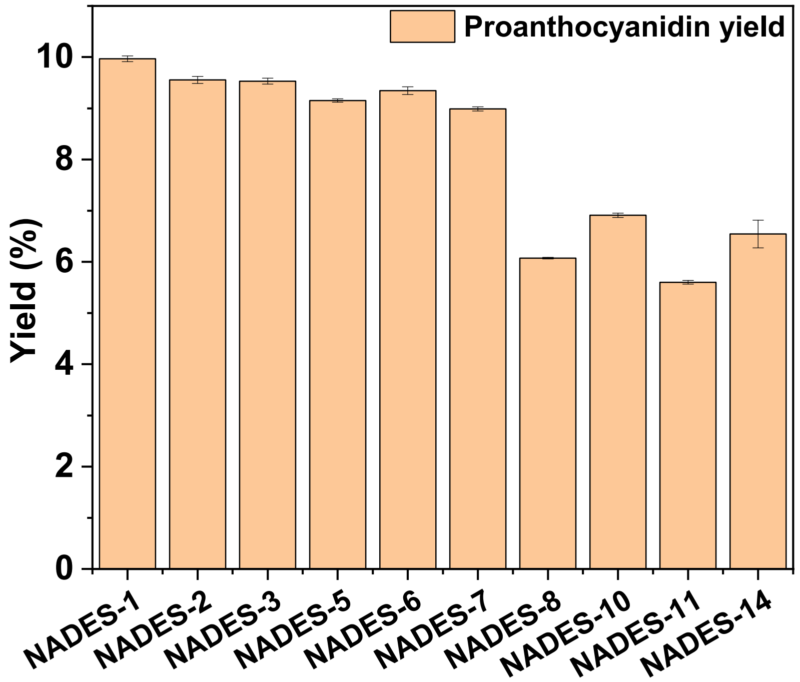

Figure S1. Effect of different NADES formulations on the extraction yield of proanthocyanidins from *Lycium ruthenicum* (Extraction conditions: solid/liquid ratio 1:15 g/mL, ultrasonic extraction at 40 °C and 240 W for 30 min).

1.3. Effect of HBA/HBD Molar Ratio

The effects of the HBA/HBD molar ratio on proanthocyanidins yield were investigated at ratios of 1:1, 1:2, 1:3, 1:4, and 1:5 (choline chloride/glycerol). The

optimal HBA and HBD molar ratio was determined based on the highest proanthocyanidins extraction yield, as shown in **Figure S2**.

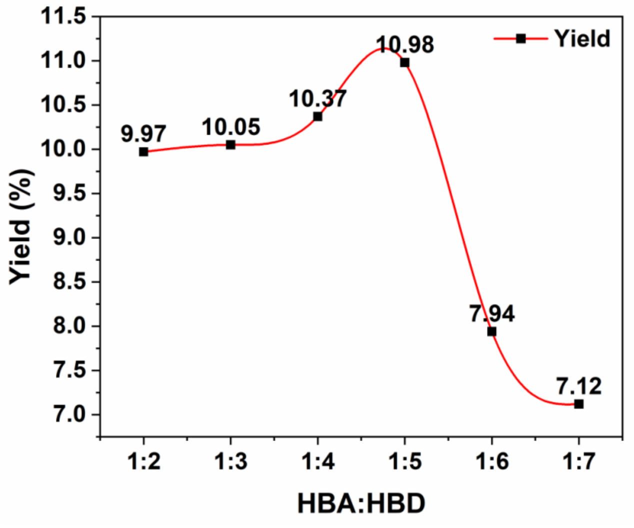

Figure S2. The extraction yield of proanthocyanidins from *Lycium ruthenicum* by NADES with different ratios of HBA and HBD.

1.4. Effect of Water Content

Using the optimal molar ratio, NADES were prepared with different water contents (0%, 10%, 30%, 50%, and 70%, v/v). The optimal water content was determined based on the highest extraction yield obtained, as shown in **Figure S3**.

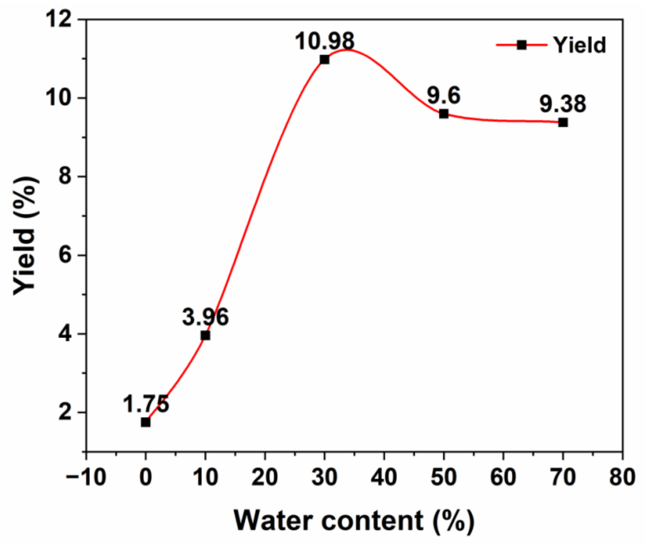

Figure S3. Effect of water content on the extraction yield of proanthocyanidins from *Lycium ruthenicum*.

From the Fourier Transform infrared spectroscopy (FTIR), the region 3000–3700 cm^{-1} corresponds to the O–H and N–H stretching vibrations involved in hydrogen

bonding. The neat NADES exhibits a very deep and broad absorption valley in this region, which is primarily attributable to the extensive hydroxyl stretching of glycerol molecules and the weak N–H stretching on the quaternary ammonium group of choline chloride. The chloride anion forms strong intermolecular hydrogen bonds with the glycerol hydroxyl groups, establishing a dense eutectic network. The high degree of hydroxyl association gives rise to extremely strong absorption with a broad, red-shifted band shape. Upon the addition of water and with increasing water content, the absorption valley intensity increases slightly, and the band shifts marginally to higher wavenumbers (blue shift). This behaviour arises from the abundant hydroxyl groups of water, which act as both hydrogen-bond donors and acceptors, competitively replacing the original glycerol–choline chloride hydrogen-bond network and generating new glycerol–water and choline chloride–water networks.

The region 2800–3000 cm^{-1} is assigned to saturated C–H stretching vibrations, originating from the methyl groups of the choline chloride quaternary ammonium and the methylene groups in the glycerol carbon chain. All five curves display a gentle, weak absorption band; upon the addition of water, only a uniform decrease in intensity is observed, with no peak shift or emergence of new peaks. This indicates that the addition of water results solely in physical dilution, leaving the alkyl backbones of choline chloride and glycerol chemically unchanged, with no chemical reactions taking place.

The 1600–1700 cm^{-1} region is mainly associated with the H–O–H bending vibration of water molecules. The neat NADES shows a flat absorption in this region, whereas the absorption gradually intensifies with increasing water content. This is attributed to the characteristic bending vibration peak of liquid water, and the absence of any new peaks further supports that water acts only as a physically mixed component.

The 1000–1500 cm^{-1} region corresponds to the C–O single-bond fingerprint area (the glycerol characteristic region). The strong absorption valley at 1050–1200 cm^{-1} arises from the C–O stretching vibration of glycerol hydroxyl groups, a hallmark peak of glycerol. A weak absorption near 1450 cm^{-1} is due to the methyl bending vibration

of choline chloride. In the neat NADES, this absorption is the strongest, as glycerol hydroxyl groups form dense hydrogen bonds with choline chloride, enhancing the C–O vibration. With increasing water content, the depth of this absorption valley progressively diminishes. Water molecules compete for the hydroxyl hydrogen-bond sites, weakening the interaction between glycerol and choline chloride anions.

The 500–1000 cm^{-1} region reflects ionic bonds and molecular skeleton vibrations of the NADES system. The most intense absorption valley in the entire spectrum appears near 1000 cm^{-1}, which is a characteristic signature of the choline chloride–glycerol NADES. It encompasses electrostatic and hydrogen-bond interactions between the anion and glycerol hydroxyl groups, along with glycerol carbon-skeleton bending and quaternary ammonium cation skeleton vibrations. As water content increases, this valley becomes considerably shallower. The intervention of water molecules screens the electrostatic interactions between quaternary ammonium cations and glycerol hydroxyl groups, thereby disrupting the ionic–hydrogen-bond composite network of the deep eutectic solvent.

Throughout the spectral range, no new characteristic absorption peaks are observed, confirming that water mixes with choline chloride and glycerol solely through physical means, without any bond cleavage or formation. All the observed changes in the system originate from three effects: hydrogen-bond competition, electrostatic screening, and physical dilution.

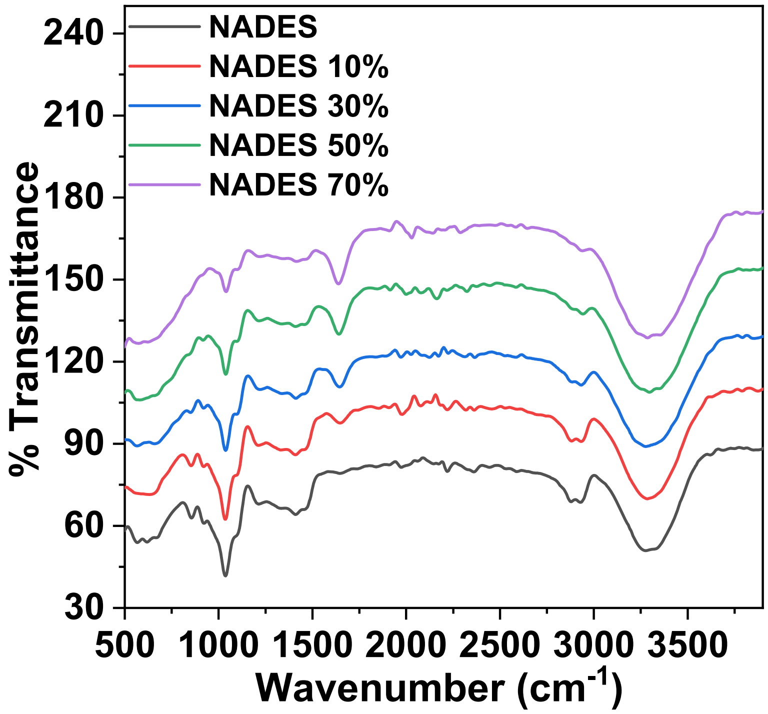

Figure S4. Fourier Transform infrared spectroscopy (FTIR) for the NADES (choline chloride /glycerol) with different water contents.

1.5. Single-Factor Experiments

Using the optimal NADES (molar ratio 1:5, 30% water content) as the extraction solvent, single-factor experiments were conducted to evaluate the effects of key ultrasonic parameters on proanthocyanidins yield. The following factors were investigated: solid/liquid ratio (1:5, 1:10, 1:15, 1:20, and 1:25 g/mL); ultrasonic power (200, 240, 280, 320, and 360 W); ultrasonic temperature (30, 40, 50, 60, and 70 °C); ultrasonic time (10, 20, 30, 40, 50 min).

1.6. Response Surface Methodology Optimization

Based on the single-factor experimental results, a central composite design (CCD) **Error! Reference source not found.** was employed to optimize the ultrasound-assisted NADES extraction conditions. Proanthocyanidins yield (*Y*) was selected as the response variable. Four independent variables were investigated: solid/liquid ratio (*A*), ultrasonic power (*B*), ultrasonic temperature (*C*), and ultrasonic time (*D*), each at five levels. The factor levels and experimental design are presented in **Table S2**. Design-Expert software was used for experimental design, data analysis, and model building.

Analysis of variance (ANOVA) was performed to evaluate the significance of the model and individual factors, and response surface plots were generated to visualize factor interactions (**Table S3**).

Table S2. Factors and levels studied using Central Composite Design (CCD).

Factors	Coded Symbols	Levels				
		-2	-1	0	1	2
Solid/liquid ratio (g/mL)	*A*	1:5	1:10	1:15	1:20	1:25
Ultrasonic power (W)	*B*	80	160	240	320	400
Ultrasonic temperature (°C)	*C*	20	30	40	50	60
Ultrasonic time (min)	*D*	10	20	30	40	50

Table S3. Experimental design and results of Central Composite Design (CCD).

**Std**	***A* (g/mL)**	***B* (W)**	***C* (°C)**	***D* (min)**	**Yields (%)**
1	1:5	240	40	30	7.42
2	1:15	240	40	30	12.22
3	1:10	320	30	20	8.19
4	1:15	80	40	30	9.13
5	1:20	160	30	20	9.56
6	1:15	400	40	30	8.49
7	1:15	240	60	30	9.23
8	1:20	320	50	20	9.92
9	1:15	240	40	30	12.31
10	1:10	160	50	40	9.93
11	1:20	160	50	40	9.82
12	1:15	240	40	30	12.20
13	1:10	160	30	20	8.24
14	1:20	160	50	20	8.85
15	1:15	240	40	30	11.70
16	1:10	320	30	40	8.46
17	1:20	320	50	40	9.81
18	1:10	160	50	20	9.53
19	1:10	320	50	20	8.29
20	1:15	240	40	10	9.84
21	1:15	240	40	30	12.21
22	1:15	240	20	30	9.66
23	1:20	320	30	20	9.87

24	1:15	240	40	50	9.99
25	1:10	160	30	40	9.58
26	1:10	320	50	40	8.32
27	1:20	320	30	40	8.53
28	1:15	240	40	30	11.89
29	1:25	240	40	30	8.29
30	1:20	160	30	40	8.87

1.7. Validation of the Kinetic Model

To validate the predictive capability of the established kinetic model, proanthocyanidin yields were experimentally determined at extraction times of 20 and 40 min for each temperature and compared with the values predicted by the Logistic model (**Table S4**). The degree of fit between predicted and measured values ranged from 0.934 to 0.995, demonstrating excellent agreement and confirming the reliability of the Logistic model in describing the extraction kinetics. These results indicate that the Logistic model effectively captures the correlation between proanthocyanidin yield and ultrasonic duration across different temperatures, providing a scientific basis for understanding temperature-dependent extraction behavior and optimizing process parameters for enhanced yield.

Table S4. Verification of extraction kinetic model of proanthocyanidins.

		Proanthocyanidins Yield (%)			
Time (min)	Parameter	20°C	30°C	40°C	50°C
20	Predicted value	8.671	8.689	8.845	9.268
	Measured value	8.848	8.875	8.893	9.928
	R^2	0.980	0.979	0.995	0.934
40	Predicted value	10.070	10.608	10.963	10.796
	Measured value	10.358	10.753	11.046	10.980
	R^2	0.972	0.987	0.993	0.983

1.8. Determination of the Actual Proanthocyanidin Content

A standard curve for proanthocyanidins was first established via HPLC. Under the optimized extraction conditions, the actual sample was then prepared. The proanthocyanidin content in this sample was subsequently determined by HPLC and

compared with the standard curve. The retention time profiles confirmed that the

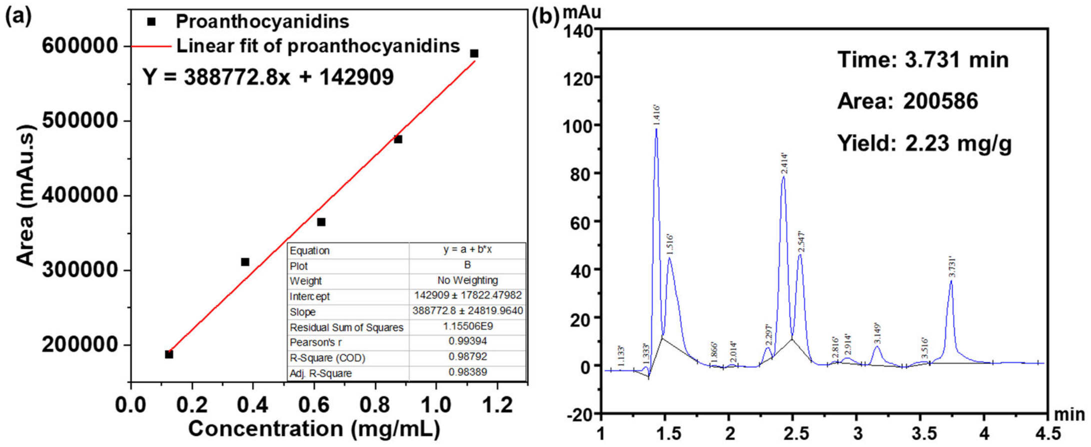

proanthocyanidin structure remained unchanged throughout the procedure. The extraction yield from the actual sample was calculated to be 2.23 mg/g, which was a 3.35% yield for proanthocyanidin (**Figure S5**).

Figure S5. (a) Standard curve of proanthocyanidins by HPLC experiments; (b) the actual extract proanthocyanidins from *Lycium ruthenicum* was tested by HPLC.

1.9. Antioxidant Activity Assays

1.9.1. Hydroxyl Radical (•OH) Scavenging Assay

The hydroxyl radical scavenging activity was evaluated according to the method described by Bors **Error! Reference source not found.** with slight modifications. Reaction mixtures were prepared by adding 1.0 mL of black goji proanthocyanidins extract at various concentrations to test tubes, followed by 1.0 mL each of 1.5 mmol/L $FeSO_4$ solution, 2.0 mmol/L salicylic acid solution, and 2.2 mmol/L H_2O_2 solution. After mixing and incubating for 30 min, the absorbance was measured at 510 nm (A_1). Control measurements were performed using distilled water instead of H_2O_2 (A_2) and instead of the proanthocyanidin extract (A_3). The •OH scavenging rate (R_1) was calculated using the following equation (4):

$$R_1 = \left[1 - \frac{A_1 - A_2}{A_3}\right] \times 100\% \quad (4)$$

1.9.2. DPPH Radical (DPPH•) Scavenging Assay

The DPPH• scavenging activity was determined following the method of Xiao et al. **Error! Reference source not found.** with minor modifications. DPPH (7.86 mg) was dissolved in anhydrous ethanol and diluted to 100 mL. The solution was stored in the dark and diluted to an absorbance of 0.4-0.8 at 517 nm before use. The

proanthocyanidins extract (2 mL) was mixed with 2 mL of DPPH solution and allowed to react in the dark for 30 min. Absorbance was measured at 517 nm (A_1). Background absorbance (A_2) was measured using anhydrous ethanol instead of DPPH solution, and blank absorbance (A_0) was measured using anhydrous ethanol instead of sample solution. The DPPH• scavenging rate (R_2) was calculated as follows:

$$R_2 = \left[1 - \frac{A_1 - A_2}{A_0}\right] \times 100\% \qquad (5)$$

1.9.3. $ABTS^{+}$• Radical Scavenging Assay

The $ABTS^{+}$• scavenging activity was evaluated using the method described by Lu et al. **Error! Reference source not found.** with slight modifications. ABTS stock solution was prepared by mixing equal volumes of 7 mmol/L ABTS and 2.45 mmol/L potassium persulfate and allowing the mixture to stand in the dark for 12-16 h. Before use, the stock solution was diluted with ethanol to an absorbance of 0.70±0.02 at 734 nm to obtain the working solution. The proanthocyanidin extract (0.8 mL) at various concentrations (0.005, 0.01, 0.02, 0.03, and 0.04 mg/mL) was mixed with 7.2 mL of $ABTS^{+}$• working solution. After 15 min of reaction in the dark, absorbance was measured at 734 nm (A_1). Blank absorbance (A_0) was measured using distilled water instead of the sample. The $ABTS^{+}$• scavenging rate (R_3) was calculated using the following equation (6):

$$R_3 = \left[1 - \frac{A_0 - A_1}{A_0}\right] \times 100\% \qquad (6)$$

1.9.4. Calculation of IC_{50}

The IC_{50} values were calculated using the equation described in *3.6 Calculation of* IC_{50} of the main text. Curve fitting was performed using GraphPad Prism 8.0 software based on the Levenberg–Marquardt algorithm. The results are presented in **Table S5**.

Table S5. Studies on the antioxidant activities of the activities extracts from various plant species.

Part of plant	Type of study	Extraction type	Method for evaluation of antioxidant activity	IC_{50} (mg/mL)	References
Quercus salicina Blume	Antioxidant capacity of phenolic contents	Ethanol (99.5%)	DPPH	0.067	Aung et al. 2020 [67]
			ABTS	0.523	
Cinnamomum camphora	Proanthocyanidins in Leaves	70% (V/V) acetone aqueous solution	DPPH	0.078	Zhai et al. 2017 [68]
			ABTS	0.117	
Cinnamomum camphora	Proanthocyanidins in Branches	70% (V/V) acetone aqueous solution	DPPH	0.274	
			ABTS	0.23	
Peanut skins crude	Proanthocyanidins	Ethanol (65%)	DPPH	0.0115	Yu et al. 2025 [69]
			Hydroxyl free radical	0.746	
Lycium ruthenicum	Proanthocyanidins	NADES (choline chloride/glycerol =1:5, 30% v/v water)	Hydroxyl free radical	0.029	This work
			DPPH	0.00056	
			ABTS	0.011	

Reference

62. Han, X.; Zhou, Q.; Gao, Z.; Xu, G.B.; Chen, H.; Chitrakar, B.; Sun, Y.S.; Zhao, W.; Lin, X.; Zhou, K.X.; Zhang, Z.S. Characteriza-tion of procyanidin extracts from hawthorn (Crataegus pinnatifida) in human colorectal adenocarcinoma cell line Caco-2, simulated digestion, and fermentation identified unique and novel prebiotic properties[J]. *Food Research International*, **2023**, *165*, 112393. https://doi.org/10.1016/j.foodres.2022.112393.
63. Delgado, M.C.; Merino, J.; Estevez, M.; Silvestre, A.J.D. Impact of eutectic solvents utilization in the microwave assisted extrac-tion of proanthocyanidins from grape pomace[J]. *Molecules*, **2021**, *27(1)*, 246. https://doi.org/10.3390/molecules27010246.
64. Bajpai, S.; Gupta, S.K.; Dey, A.; Jha, M.K.; Bajpai, V.; Joshi, S.; Gupta, A. Application of central composite design approach for removal of chromium (VI) from aqueous solution using weakly anionic resin: Modeling, optimization, and study of interac-tive variables[J]. *Journal of Hazardous Materials*, **2012**, *227-228*: 436-444. https://doi.org/10.1016/j.jhazmat.2012.05.016.
65. Xiao, J.; Li, S.Y.; Sui, Y.; Li, X.P.; Wu, Q.; Zhang, R.F.; Zhang, M.W.; Xie, B.J.; Sun, Z.D. In vitro antioxidant activities of proan-thocyanidins extracted from the lotus seedpod and ameliorative effects on learning and memory impairment in scopola-mine-induced amnesia mice[J]. *Food Science and Biotechnology*, **2015**, *24(4)*: 1487-1494. https://doi.org/10.1007/s10068-015-0192-y.
66. Lu, D.; Wang, L.J.; Zhang, W.; Guo, B.H.; Lv, Y.G. Study on the antioxidant ability of procyanidins and their complexes[J]. *E3S Web of Conferences*, **2022**, *341*: 01013. https://doi.org/10.1051/e3sconf/202234101013.
67. Aung, T.; Bibat, M.A.D.; Zhao, C.C.; Eun, J.B. Bioactive compounds and antioxidant activities of Quercus salicina Blume extract. *Food Sci. Biotechnol.*, **2020**, *29*, 449–458. https://doi.org/10.1007/s10068-020-00755-1.
68. Yang, H.B.; Xu, P.L.; Song, W.; Zhai, X.Q. Anti-tyrosinase and antioxidant activity of proanthocyanidins from Cinnamomum camphora. *International Journal of Food Properties*, **2017**, *24(1)*, 1265–1278. https://doi.org/10.1080/10942912.2021.1958841.
69. Yu, L.; Feng, S.; Song, Y.; Bi, J.; Gao, Y.; Wang, L.H.; Jiang, C.; Wang, M.Q. Exploring the extraction, antioxidant activities and its stabilities of peanut skins crude proanthocyanidins extract. *Sci. Rep.*, **2025**, *15*, 26274. https://doi.org/10.1038/s41598-025-10934-x.
